# Novel 1-bit hybrid reconfigurable intelligent surface

**DOI:** 10.1038/s41598-026-55424-w

**Published:** 2026-06-08

**Authors:** Sajedeh Keshmiri, Suren Jayasuriya, Mohammadreza F. Imani

**Affiliations:** School of Electrical, Computer, and Energy Engineering, Arizona State University, Tempe, AZ 85287 USA

**Keywords:** Engineering, Mathematics and computing, Physics

## Abstract

Reconfigurable intelligent surfaces (RISs) are anticipated to play a key role in future smart wireless networks by enabling control over the propagation environment and improving communication performance. However, most existing RIS designs rely on external channel information acquisition, thereby restricting their autonomy. By embedding sensing directly into the RIS, the resulting hybrid RIS (HRIS) can pave the way for a self-configuring wireless network. However, previous HRISs used complex geometries, resulting in low sensing signal strength or costly implementation. This paper proposes a novel 1-bit HRIS designed to sense the incident signal’s angle of arrival (AoA) and redirect it toward desired directions. This device consists of independently tunable resonant patch elements loaded with PIN diodes. To introduce sensing capabilities, a portion of the incident signal on each element is coupled into a parallel-plate waveguide (PPWG) via small rectangular slots. This PPWG is below the reconfigurable patch array of the HRIS and collects the coupled signal from all elements. Two coaxial connectors are then used to sample the signal coupled to the PPWG. We use computational processing and a multilayer perceptron (MLP) to analyze signals collected in this manner to detect AoA. Further, pre-coded phase randomization is implemented by varying slot sizes to suppress undesired quantization lobes. The proposed HRIS is simple and low-cost, and it can pave the way for intelligent wireless communication, power transfer, and sensing without requiring feedback loops.

## Introduction

Reconfigurable intelligent surfaces (RISs) consist of tunable elements that can dynamically redirect electromagnetic waves to control the propagation environment. They can optimize signal paths, mitigate interference, and enhance network security^1–14^. To effectively tailor the propagation environment, RISs require information about desired directions. However, this requirement is often overlooked in the current literature on RIS-empowered wireless networks, which typically assumes that the full or partial channel state information is readily available. Instead, the focus has been on designing reflecting metasurface configurations that can achieve the desired reconfigurable reflection patterns, or investigate their capabilities in improving wireless links^11–15^.

Over the years, various methods for obtaining channel state at the RIS have been proposed. The primary method is to measure the BS-RIS-UE (Base Station-RIS-User Equipment) channel and use that to decompose the separate channels of BS-RIS and RIS-UE^16–19^. This method is computationally expensive and susceptible to noise. In many practical scenarios, RISs do not require the full CSI information. Approximate angular ranges of the transmitter and user(s) may be sufficient to redirect signals toward desired directions (or mitigate interference)^20–22^. To this end, some works have proposed using optical cameras or radars to guide RISs^23,24^. However, such methods can significantly increase the overall system’s complexity. Ideally, we want the RIS itself to sense the required angles (using wireless signals) and adjust its pattern accordingly. Toward this goal, space-time coding metasurfaces have been proposed that can estimate the angle of arrival (AoA) of signals from measured frequency harmonics. However, this adds complexity due to the need for temporal modulation^25^.

**Fig. 1:**
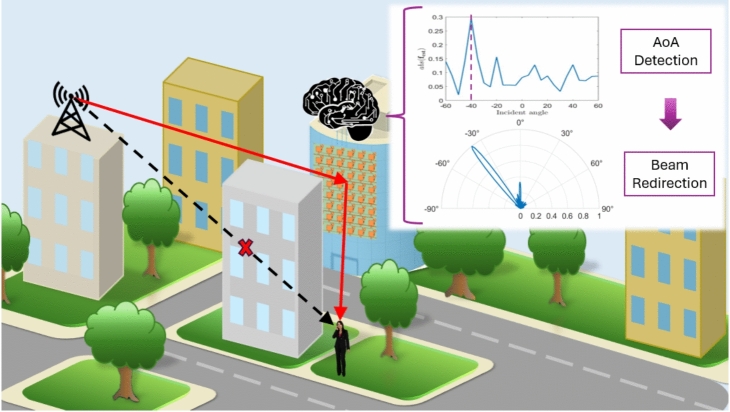
Conceptual illustration of the operation of HRIS in wireless networks. It uses random patterns and computational techniques to estimate the AoA and redirect the reflected beam to avoid blockages. Figure created by the authors using Microsoft PowerPoint (Version 2511).

A different strategy is to introduce a sensing mechanism to the RIS (Joint sensing and communication). One straightforward approach is to dedicate certain RIS elements exclusively to channel sensing, which, while effective, results in lower spatial resolution and discontinuous reflective phase profiles^26,27^. Recent works have also explored stacked intelligent metasurfaces (SIMs), where multiple programmable metasurface layers enable wave-domain signal processing, including beamforming and two-dimensional AoA estimation. These approaches demonstrate enhanced functionality through multilayer architectures and optimized phase control, but at the cost of increased system complexity and implementation challenges^28,29^. A middle ground approach is hybrid RISs (HRISs)^9,30–33^ where sensing capabilities are integrated into RISs configuration by modifying all or a few of the constituent meta-atoms to couple a portion of the incident signal into a waveguide for sampling and analysis (hybrid elements). Using the collected signal, the AoA or channel state information can be retrieved, promising a way toward a fully autonomous and smart wireless network in which the benefits of RISs are fully realized. A conceptual overview of this idea is illustrated in Fig. 1. It shows a high-level example of an HRIS assisting a blocked wireless link. By sensing the direction of the incoming signal (without requiring a prior knowledge of the base station or the receiver), the HRIS autonomously redirects the reflected beam toward the user, restoring coverage without requiring additional channel estimation or feedback.

To implement HRISs, mushroom structures with varactor diodes have been used to achieve reconfigurable resonance, with incident waves coupled to substrate-integrated waveguides (SIWs) in the bottom layer through annular slots^9^. Although this design successfully integrated sensing and reflection within a single configuration, it required many RF chains for information extraction, significantly increasing system cost and complexity. A later refinement used only a few hybrid elements connected to SIWs to reduce complexity while enabling AoA detection through sparse sampling^34^. The core concept underlying this HRIS was later experimentally demonstrated in a follow-up study^35^. However, this device relied on multiplexing the incident wave across HRIS’s elements before reaching the sensing waveguide. This led to weak signal strength, as the SIWs operated near cut-off due to space constraints. Furthermore, implementing SIWs and the required DC circuitry on the same substrate remains highly complex, increasing fabrication costs.

In this paper, we aim to develop a simple, low-cost HRIS. To do that, we propose using a 1-bit configuration with large element spacing and a parallel-plate waveguide (PPWG) to collect the sensed signal. Using 1-bit tuning with large element spacing (*~λ /2*) reduces the overall cost and power consumption of RISs, especially as the number of elements grows. However, it can cause unwanted quantization lobes that reduce efficiency and lead to interference in undesired directions^36^. In other words, there is an inherent trade-off in HRIS design. While prior HRIS implementations employing multi-bit or continuously tunable elements can achieve improved beamforming performance, they typically require additional hardware such as digital-to-analog converter (DAC) circuitry, increasing system complexity. In contrast, the proposed design adopts a simple 1-bit architecture, offering a significantly reduced hardware footprint at the cost of reduced beamforming capability. A practical solution to mitigate quantization lobes is phase randomization^36,37^, which involves introducing random phase delays in each unit cell. These random phase delays can disrupt the periodic patterns (formed by 1-bit tuning) that give rise to quantization lobes. This method effectively suppresses the grating lobes while preserving the simplicity of a 1-bit RIS.

The sensing configuration design involves two key decisions: the sensing layer and the number of sampling points. Using elements spaced farther apart than in previous HRIS designs reduces element-to-element coupling. Earlier designs used this coupling to multiplex the signal incident on all elements into the signal collected by a few hybrid meta-atoms^34^. As the spacing between elements increases, the signal collected at a hybrid meta-atom becomes dominated by the incident wave directly on that element. This can degrade the sensing process and increase detection errors. To enhance the strength of the sensing signal collected across the entire RIS while avoiding the complexity associated with multilayer or structures with blind vias (e.g. SIWs), we propose replacing SIWs with PPWGs. To allow coupling to this waveguide, we add small slots in the ground plane of each patch. This design ensures that some of the signal incident on each element are coupled to the PPWG for sensing purposes. These slots also serve another purpose: Mitigating quantization lobes due to large element spacing and 1-bit tuning. Specifically, we implement phase randomization by randomly varying the size of the coupling slots along the HRIS.

Once the sensing layer is selected, the next step is to sample the collected signal using a limited number of connectors. Similar to previous work^38^, we also employ two coaxial connectors to sample signals. Using more connectors may improve performance by providing measurement redundancy, thereby enhancing noise robustness and sensitivity; however, this comes at the cost of increased system complexity. Therefore, it is not considered in this work and is left for future studies. To utilize information from only two connectors to detect AoA from electrically large HRISs, we outline two different computational techniques: least squares solvers and a multilayer perceptron (MLP) to retrieve AoA. The main considerations in the HRIS design process are summarized in Table 1, which compares different configurations.

Preliminary results confirming this novel design were reported by Keshmiri et al.^39^. In this paper, we expand upon that work by outlining the design considerations for the proposed HRIS. Using full-wave simulations, we detail how changing slot sizes alters the effective reflective phase of the element. We will then illustrate the process to use these varied reflected phases to mitigate quantization lobes. Using full-wave simulations, we demonstrate the proposed HRIS’s ability to steer beams in desired directions without encountering quantization lobes. We also evaluate the system’s performance in detecting the AoA using different methods as a function of signal-to-noise ratio (SNR) and number of measurements.

**Table 1:** Comparison of different HRIS designs in terms of different design considerations.

References	No. of sampling points	No. of hybrid elements	Complexity	Tuning	Element spacing
Alamzadeh et al.^9^	All elements	All elements	High	Continuous	*λ /4*
Movahediqomi et al.^33^	All elements	All elements	Very high	Continuous	*λ /10*
Alamzade and Imani^34^	1 per every 7 elements	1 per every 7 elements	High	Continuous	*λ /4*
Alamzade and Imani^38^	2	2	Medium	Continuous	*λ /4*
Alamzade and Imani^35^	2	2	Medium	Binary	*λ /3*
This work	2	All elements	Low	Binary	*λ /2*

## Methods

### Metasurface element design

**Fig. 2:**
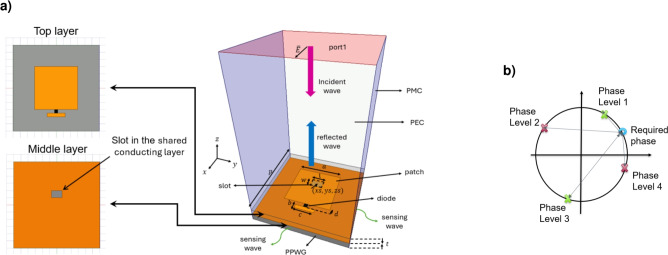
(**a**) Simulation configuration of the building block of the proposed HRIS. $(x_s, y_s, z_s) = (-3, 0, -1.624)$ which is a vector from the patch’s center to the slot’s center. Port 1 is de-embedded to the surfaces of the patch. (**b**) Quantization of the required reflection phase by selecting the closest discrete phase level in the complex domain.

The configuration of the building block for the proposed HRIS is illustrated in Fig. 2a. It features a square patch element with a side length of 12 mm, implemented on a Rogers 4003 substrate with a thickness of 1.624 mm and a relative permittivity ($\epsilon _r$) of 3.55. This element is loaded with a PIN diode, which is modeled in its *off* state as an effective capacitor of 0.15 pF and in its *on* state as a resistor of 0.1 *Ω* (similar to BAR5002VH6327XTSA1 PIN Diode, Infineon Technologies). The patch element shares a ground plane with a PPWG made from the same substrate material and thickness. A small rectangular slot is incorporated into the shared ground plane to allow a portion of the incident wave to couple into the PPWG. The geometry of this element was designed for operation at 5.6 GHz using full-wave simulations in Ansys HFSS. The resulting dimensions are reported in Table 2. While the design was specifically tailored for this frequency, it can be easily adapted to other frequencies by adjusting the geometry or the switchable component. As shown in Fig. 2, we placed the slot off-center to couple to the PPWG based on a parametric study in Ansys HFSS (not shown for brevity). The off-center placement is required as the electric field (or voltage) is usually minimum at the center of a patch antenna. A similar idea has been pursued in the literature^34^. The slot length, *l*, has a significant effect on performance and will be used later for controlling the reflection phase. The slot width, *w*, due to polarization of the electric field, does not significantly affect performance unless it becomes relatively large. Therefore, we selected a sub-wavelength *w* to avoid unwanted resonances.

**Fig. 3:**
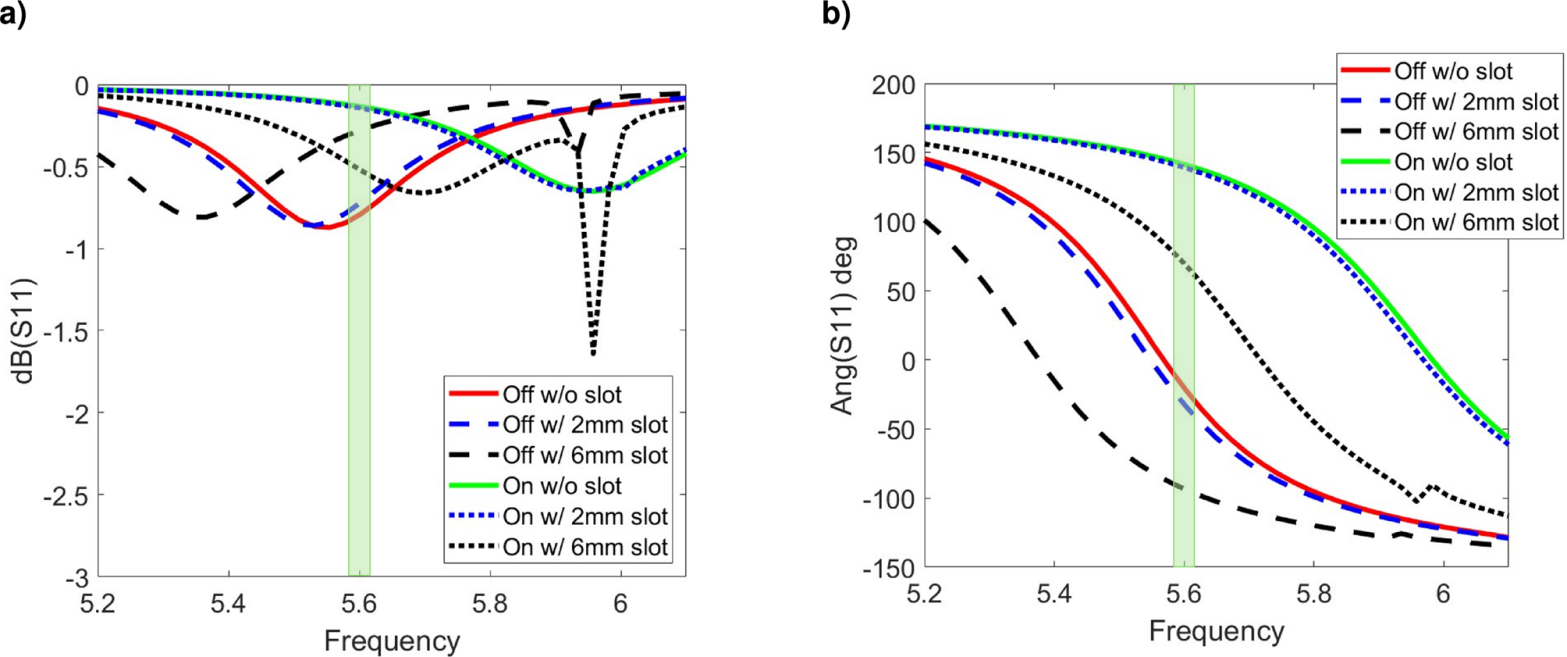
(**a**) Amplitude and (**b**) de-embedded phase of the reflection coefficient of the proposed building block.

The simulated reflection coefficient for the element, both with and without the slots, is depicted in Fig. 3. The results indicate that adding the slots does not significantly change the reflection magnitude, suggesting that only a small portion of the signal is coupled to the PPWG. In these simulations, the slot was offset by -3 mm from the substrate center in the x-direction. To use this element for beamforming without causing grating lobes, we need to increase the diversity of reflected phase. The minimum design requirement is to introduce an additional geometrical state, resulting in two distinct slot lengths. The slot behaves as a resonant element that couples a portion of the incident field into the lower layer and perturbs the reflection phase. Increasing the slot length shifts its resonance toward that of the patch, leading to a stronger influence on the reflected phase from the patch. However, when the slot length becomes comparable to the guided wavelength in the substrate, the slot and patch can strongly interact, forming coupled resonances within the same frequency band. This coupling can significantly alter the element response and disrupt the intended operating behavior. Based on this physical insight, two slot lengths were selected: 2 mm ($\approx 0.07\lambda _g$), where the slot resonance is sufficiently separated from the patch resonance, and 6 mm ($\approx 0.21\lambda _g$), where the slot resonance is closer but still avoids strong coupling. This configuration produces four reflection states corresponding to the two diode conditions (ON/OFF) and the two slot geometries. The final choice of slot lengths is determined by achieving approximately $90^{\circ }$ phase separation between these four states while maintaining low coupling to the sensing layer. As shown in Fig. 3b, slot lengths of 2 mm and 6 mm nearly reach this condition at 5.6 GHz. These dimensions provide sufficient phase diversity for adequate randomization while keeping the coupled power within an acceptable range for reliable AoA detection, as further analyzed in the *Results* section.

**Table 2:** Design parameters for the metasurface element.

Parameter	Value	Parameter	Value
a	12 mm	b	1 mm
c	5 mm	d	1 mm
l	2 or 6 mm	p	24 mm
t	1.624 mm	w	2 mm

### Beamforming

**Fig. 4:**
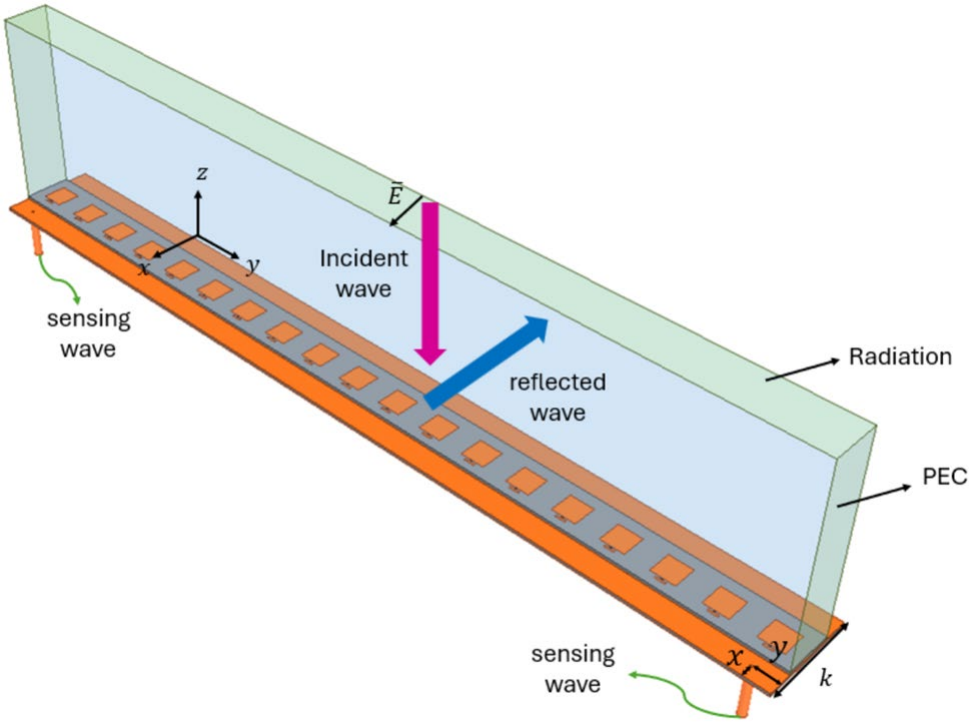
Simulation configuration of an array of 19 elements made of the proposed building block. Here, $x=6\,\text {mm}, y=12\,\text {mm}, k=48\,\text {mm}$.

Beamforming with the proposed 1-bit HRIS requires addressing two coupled design challenges. First, the limited reflection phase values and the large *λ /2* element spacing inherently lead to strong quantization lobes. We can overcome this challenge by utilizing elements with slots of different sizes. However, the choice of distribution of slot sizes to create phase randomization is related to the distribution of the diode on/off states (referred to as masks for brevity) to approximate the desired progressive phase profile for beamsteering. Because these two design knobs govern the reflected pattern together, we utilize an iterative process. To keep the problem simple, we analyze the beamforming capability of the proposed HRIS by simulating a 1D configuration consisting of 19 of the proposed elements (Fig. 4). In this manner, the beamforming and sensing capabilities are evaluated only in the yz-plane, with an infinite periodic array assumed in the perpendicular direction.

**Fig. 5:**
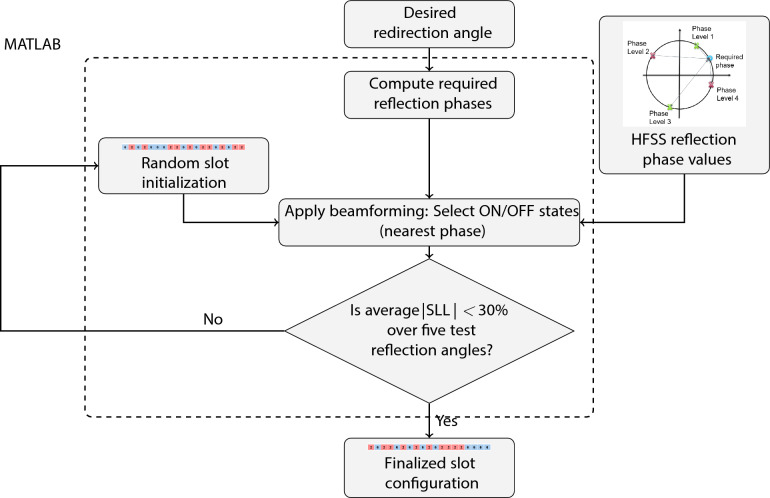
Flowchart of the beamforming and slot–selection process including the decision loop.

As shown in Fig. 5, the first step is to determine the phase gradient needed to steer the beam toward a given direction^1^. We need to use the on/off reflection phases such that the resulting phase gradient best approximates the desired gradient phase profile. However, we need first to select the slot length for each element. Specifically, to add random phase delays to the elements, each element may be assigned a slot length of 2 mm or 6 mm, which would result in different reflection phases in the on and off states. Based on HFSS simulations, each slot length supports two distinct reflection phases depending on the on/off diode state (Fig. 3b). It is worth noting that when selecting on and off states of each element to approximate the desired gradient phase profile, we aim to minimize the Euclidean distance in the complex domain between the required phase for each element and the available reflection phase obtained from the element simulations (see (Fig. 3b). Since loss is not considered in this work, this condition reduces to choosing the angle closest to the required one.

**Fig. 6:**
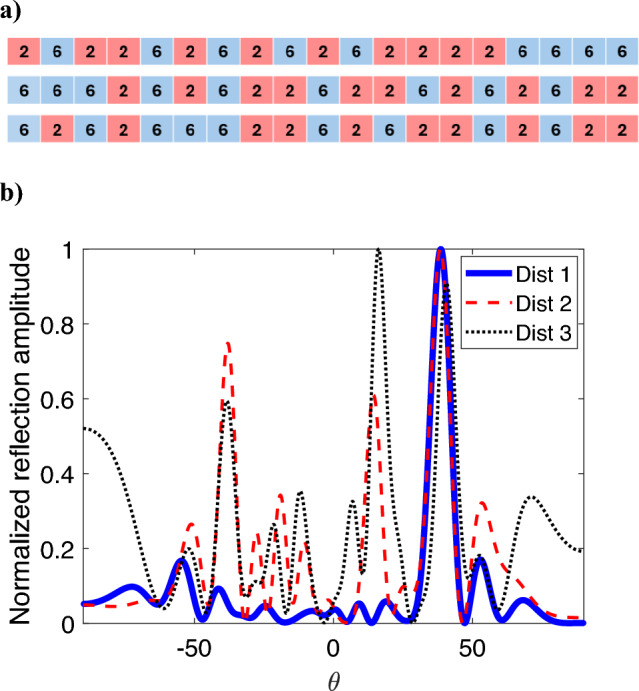
(**a**) 3 different layouts of the 1D array where elements with $2\,\text {mm}$ long slots and those with $6\,\text {mm}$ long slots are denoted by red and blue, respectively. (**b**) Numerically calculated reflection pattern for different slot distributions depicted in (**a**).

These discrete phase shifts were extracted and used in a simplified array-level simulation in MATLAB. In these numerical studies, each element is replaced by a dipole whose phase will depend on the corresponding element’s on/off status and the slot length. By calculating the corresponding array factor, we can examine the predicted quantization lobe. We used this array calculation to estimate the far-field reflection pattern for different distributions of 19 elements with 2 mm or 6 mm slots. To find an effective configuration (with minimal grating lobes), we generated several random slot-assignment distributions and simulated the corresponding far-field patterns at different reflection angles. Our selection criteria were the level of the quantization lobe over multiple redirection angles. Figure 6 shows examples of this calculation, including the one ultimately selected based on its low side lobe levels.

It is important to note that this distribution is not optimal. The optimal slot-size distribution is dictated by the optimum phase randomization and masks. In this work, we used only two slot sizes for simplicity; however, many different slot sizes (and consequently, phase randomization levels) can be employed, and their distribution can affect overall performance. Optimizing the phase randomization (and consequently, the slot-size distribution) is beyond the scope of this paper and will be addressed in future research. For this proof of concept, we utilize the distribution obtained through trial and error.

### Sensing

Once the distribution of slots has been determined, we examine the sensing capabilities of the designed HRIS. To minimize complexity, we implement AoA detection using only two coaxial connectors to collect the sensing signal.

**Fig. 7:**
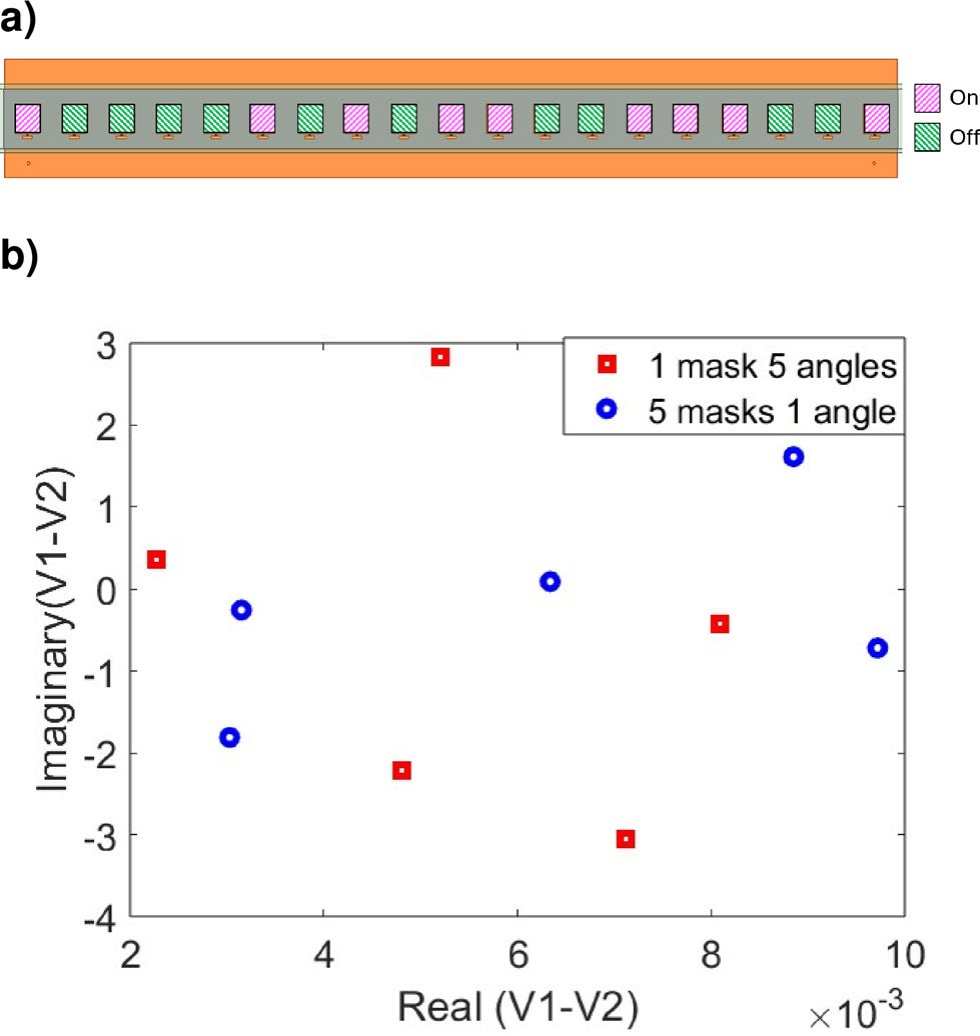
(**a**) Example of on/off (pink and green) distribution of elements. (**b**) Difference in the voltages induced on the coaxial connectors due to incident signals with five different incident angles for the mask in (**a**), and for the case of one incident angle with five different random masks.

Using this simulation setup, we now examine the signal collected by the coaxial connector and devise methods to detect AoA. Figure 7b illustrates the differences in voltages induced on the coaxial connectors as the AoA changes. This observation confirms that the signal measured at the coaxial connectors contains information regarding the AoA. However, this relationship is nontrivial and cannot be inverted easily. Additionally, more measurements are required to determine the AoA accurately. To address this issue, we utilize random masks. As shown in Fig. 7b, applying different masks for each AoA yields new measurements, improving the condition number of the inverse problem.

**Fig. 8:**
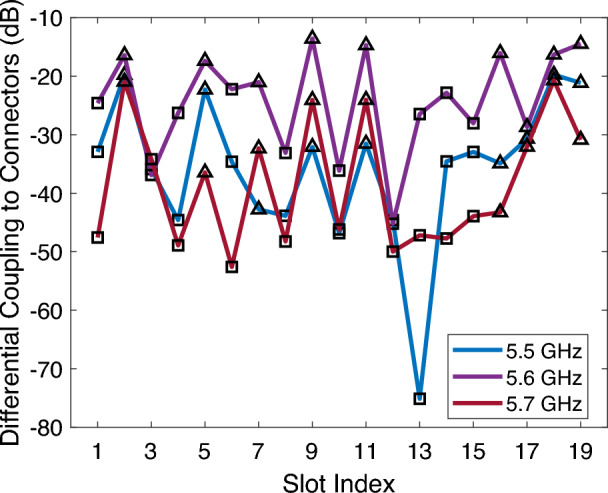
Differential coupling from the slots to the connectors at three frequencies. Slots of 2 mm and 6 mm lengths are indicated by square and triangular markers, respectively.

To retrieve AoA from the multiplexed signal shown in Fig. 7b, we utilize two different methods:

(1) In the first method, we follow a process similar to that previously reported^34^. We start by discretizing the possible AoAs into *N* reference angles, ranging from $-60^\circ$ to $60^\circ$ in $5^\circ$ increments, resulting in 25 reference angles. The sensing matrix, $\mathbf{H}_{M \times N}$, is then constructed by collecting the difference between the voltages on the coaxial connectors caused by incident waves arriving from reference angles using *M* masks with random on/off distribution. The estimation problem then follows the formulation:

1 $$\begin{aligned} \mathbf{g}_{M \times 1} = \mathbf{H}_{M \times N} \mathbf{f}_{N \times 1}, \end{aligned}$$

where $\mathbf{g}$ represents the measurements for an arbitrary incident signal. The ith element of $\mathbf{f}$ is one if the incident AoA coincides with the ith reference angle and zero otherwise. Since $\mathbf{H}$ is not square, we use a computational technique (i.e. Conjugate Gradient Squared (CGS)) to estimate $\mathbf{f}$. The resulting estimated vector, $\mathbf {f_{est}}$, would not be all 0s and 1s. To deduce the AoA, we consider the index of the maximum of $|\mathbf {f_{est}}|$ to be the estimated AoA. If the incident signal does not align with a reference angle, the closest reference angle is used for the estimation.

In the configuration shown in Fig. 4, we have used connectors that are near the edges to ensure that no single element dominates the collected signal. However, we expect sensing performance to remain largely unchanged for different connector placements. This is because the PPWG behaves like a cavity that collects and mixes the signals coupled from all elements. As a result, the measured signal represents a multiplexed combination of contributions from the entire array, rather than being dominated by nearby elements. To further illustrate this point, we plotted the coupling between different slots and the coaxial connectors in Fig. 8. This figure was generated by simulating the ppwg layer, with the slots replaced by waveports of the same size. Since we use the difference between signals collected at the coaxial connector, we also examine the difference between the signals transmitted from the slots to the connectors, which is distributed across the array and is not dominated by any single slot. We studied this phenomenon at two nearby frequencies that demonstrate similar performance but exhibit distinct characteristics. The significant difference between adjacent frequencies is a notable feature of the ppwg cavity. One can also view this in another way: the differences between adjacent frequency points are analogous to moving the coaxial connector. As observed, while certain variations occur, the overall signal behavior remains similar and is not dominated by any single slot.

(2) In the second method, we used a neural network-based approach using a multilayer perceptron (MLP) classifier. The same set of 25 reference incident angles used for CGS was used to construct the training data, where each sample consisted of 16 features representing the real and imaginary parts of the voltages measured across four distinct masks. To improve generalization, the training dataset was synthetically expanded through noise-based augmentation. Specifically, 30 noisy realizations of each original sample were generated by adding Gaussian noise with signal-to-noise ratios (SNRs) randomly drawn between 10 dB and 30 dB. This resulted in a 30-fold increase in the size of the training dataset and enabled the model to learn robustness to measurement noise. The MLP architecture consisted of two hidden layers with ReLU activations and dropout regularization.

The Gaussian noise considered in this work is physically grounded in thermal noise at the receiver and represents the dominant noise source in the system. Cross-coupling within the PPWG is inherently captured in $\mathbf{H}_{M \times N}$, as the connectors record the combined contribution from all elements. Similarly, coupling between the connectors is also embedded in these responses and does not require separate modeling. External interference is not explicitly considered. In cases where multiple users share the same spectrum, it is necessary to distinguish between the primary and the secondary users. This can be achieved by incorporating additional receiver capabilities (e.g., demodulation) to enable the system to identify and track intended users and suppress interference (e.g., through null formation)^22^. Such extensions would transform the system into a more intelligent and adaptive communication platform and are left for future work.

## Results

### Beamforming

**Fig. 9:**
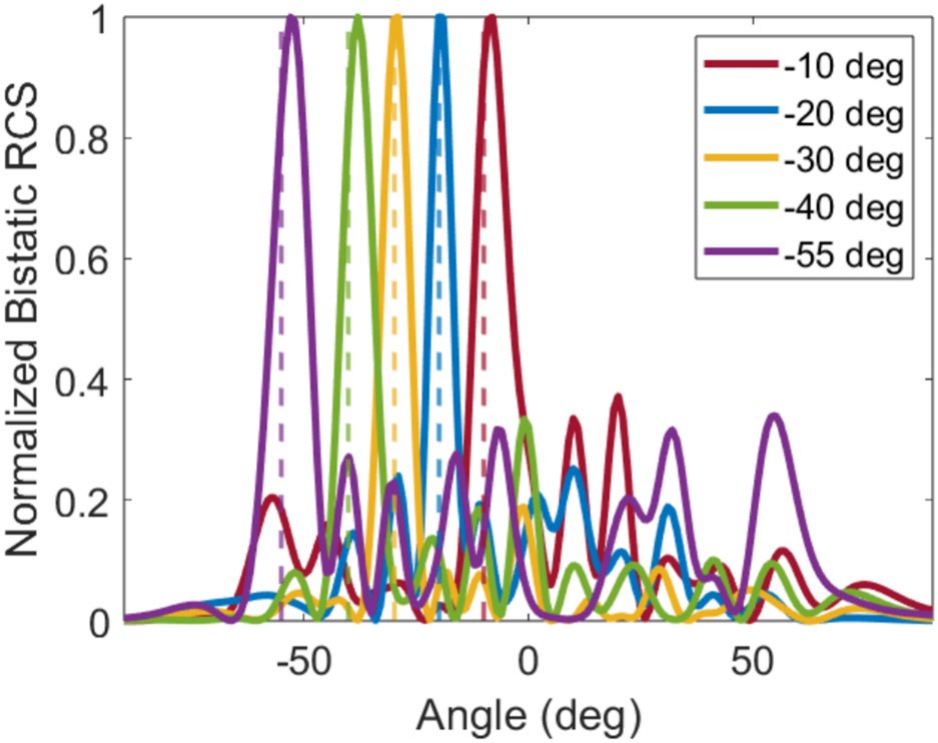
Beamforming results for five different desired angles (denoted by dashed lines), assuming a normal incident wave.

Figure 9 presents the simulated reflection patterns of the proposed HRIS for several desired beam steering angles, obtained using the simulation configuration described in the “Methods” section. The results confirm that the HRIS can effectively redirect a normally incident wave toward different prescribed directions while maintaining a well-defined main lobe and suppressed quantization lobes. The introduction of slot-based phase randomization disrupts the periodicity of the reflected phase profile, thus mitigating the strong grating lobes commonly observed in conventional 1-bit RISs with large element spacing.

It is worth noting that, although a periodic infinite boundary condition is applied in the perpendicular direction in this study, a practical two-dimensional (2D) array is finite and therefore subject to edge effects. This issue is common to all RIS designs and is typically mitigated by using larger arrays. In this case, a 2D HRIS can be realized by tiling multiple subarrays. The number of tiles and their size depend on the particular application and should be optimized while accounting for fabrication constraints. It is also worth noting that, as shown in Alamzadeh and Imani^35^, since the sensing matrix is constructed from experimental measurements, it inherently captures edge effects, and the sensing performance is not impacted by finite size.

### Sensing

**Fig. 10:**
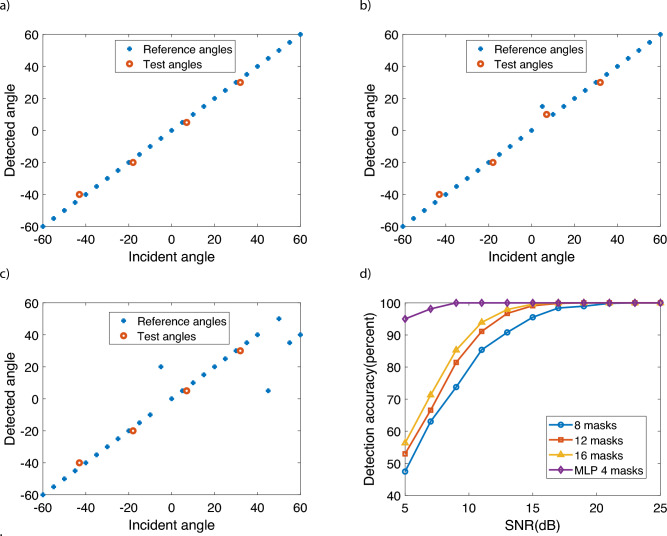
Detected versus the actual AoA for the case with (**a**) 16 masks and SNR=20 dB, (**b**) 16 masks and SNR=15 dB, (**c**) 8 masks and SNR=15 dB, and (**d**) detection accuracy for different SNR levels and number of masks, averaged over four distinct test angles:[$-43^\circ$ $-18^\circ$ $7^\circ$ $32^\circ$].

The sensing performance of the proposed HRIS was evaluated using the AoA detection framework described in the “Methods” section. Figure 10a–c presents the detected angle versus the actual incident angle, including reference angles used to form the sensing matrix and additional test angles ($-43^\circ$ $-18^\circ$ $7^\circ$ $32^\circ$) not included in the reference set for different SNR levels and different mask numbers. The results demonstrate that the proposed HRIS system can estimate AoA with an accuracy of $\pm 3^\circ$ (when *M=16* and SNR=20 dB, shown in Fig. 10a). In Fig. 10b, the SNR level is reduced to 15 dB. A slight degradation in estimation accuracy can be observed. Fig. 10c shows the results with only 8 masks at an SNR of 15 dB. In this case, the estimation performance noticeably decreases, indicating that a higher number of spatial masks is beneficial for maintaining accuracy under low-SNR conditions when using the CGS method.

We conducted Monte Carlo simulations to evaluate the detection accuracy for different scenarios more systematically. For each SNR value, 50 independent trials with new instances of the added noise were performed. In each trial, the AoA was estimated using the proposed sensing method. The detection was considered successful if the estimated angle was within $\pm 3^\circ$ of the actual incident angle. The average detection accuracy across the 50 trials was calculated to generate each data point in Fig. 10d. This process was repeated for configurations with 8, 12, and 16 masks. Fig. 10d shows that a larger number of masks (12 or 16) yields better performance (than 8 masks) at low to moderate SNRs; however, the effect of mask number diminishes as the SNR exceeds 20 dB, where all configurations approach nearly 100% accuracy.

We conducted a similar study for the MLP method using the same Monte Carlo procedure. The trained model was evaluated on the same test angles used for the CGS method. The classification was repeated 50 times for each SNR value with independently generated noise samples added to the test. Detection accuracy was calculated in a similar manner as above. The results of this study is shown in Fig. 10d. The multilayer perceptron classifier maintains high classification accuracy across the entire SNR range, even when trained using measurements from only four masks, confirming the robustness of the proposed HRIS for AoA detection under various signal conditions.

In this work, random masks were used to construct the sensing matrix and perform AoA detection. This approach has been shown to be sufficient for constructing a well-conditioned sensing matrix and achieving reliable AoA detection^40,41^. However, optimized mask designs could further improve efficiency, for example, by reducing the number of required measurements or enhancing reconstruction accuracy. One approach would be to use masks that generate (nearly) orthogonal reception patterns^42^. Additionally, the mask selection process could be incorporated into a learning-based framework in which the masks and the neural network are jointly optimized^43^. These directions offer promising opportunities for enhancing system performance, but are beyond the scope of this work and are left for future investigation.

The proposed approach can be extended to 2D AoA detection by defining the sensing matrix over both azimuth and elevation angles. The same measurement process can be employed; however, a larger number of measurements is required to resolve both angular dimensions. For 2D detection, the main practical challenge is the independent DC biasing of a large number of elements, especially if a multilayer structure is to be avoided. As the array size increases, routing becomes more complex. A practical solution is to partition the HRIS into smaller sub-panels, each controlled by a local microcontroller (MCU) or shift register. This modular approach simplifies biasing and enables scalable implementation.

### HRIS signal strength analysis

To quantify the level of signal coupled to the sensing layer (ppwg), we compared the total power passing through the slots to the total incident power for different mask configurations. During the beamforming phase, the average coupled power accounted for approximately 2.8% of the total incident power, indicating that only a negligible fraction of the impinging energy is diverted from the main reflection path. This confirms that the slots have minimal impact on beamforming performance and overall radiation efficiency. In the AoA detection phase, where random masks are applied, the average power coupled through the slots is approximately 4.6% of the total incident power. This small fraction of the signal is sufficient for an accurate angle estimation. These results verify that the proposed HRIS can perform reliable sensing with minimal energy loss from the primary reflective channel.

### Statistical evaluation of slot configurations

**Fig. 11:**
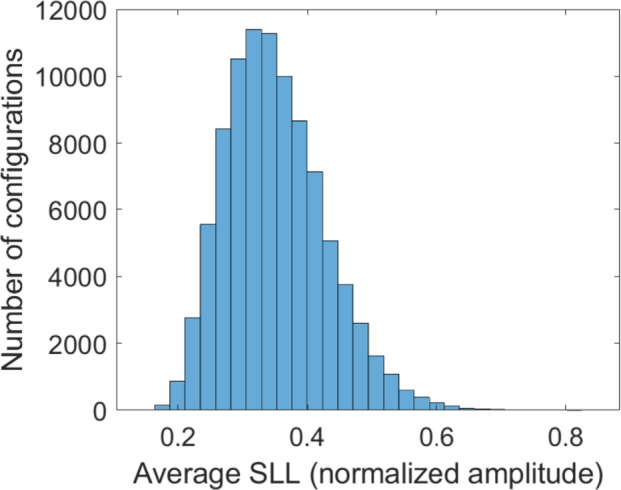
Distribution of the average (over five reflection angles) SLL across all unique slot configurations, demonstrating that the reported configuration represents a common solution with an average SLL below 0.3.

We designed our HRIS to suppress grating lobes following a similar trial-and-error procedure used in previous work to realize phase randomization for quantization-lobe suppression^36,37^. While satisfactory results can be achieved, the question is how readily the desired performance can be attained and whether higher performance is possible. To more systematically evaluate the impact of slot distribution on beamforming performance, we conducted an exhaustive analysis using a MATLAB simulator over all possible configurations of a 19-element array, where 10 elements are assigned 2 mm slots and the remaining 9 elements use 6 mm slots. This results in a total of $$\left( {\begin{array}{c}19\\ 10\end{array}}\right) =92{,}378$$ unique configurations. To evaluate performance under different operating conditions, each configuration was analyzed for five desired reflection angles: $10^\circ$, $20^\circ$, $30^\circ$, $40^\circ$, and $50^\circ$. For each angle, the normalized far-field radiation pattern was obtained (using the MATLAB simulation described in the Beamforming subsection of the Methods section), and the SLL was extracted. The average SLL across these angles was used as a performance metric. The distribution of the average SLL across all configurations is illustrated in Fig. 11. The results indicate that configurations achieving an average SLL below 0.3 (normalized amplitude), corresponding to approximately 10 dB, are the most probable within the design space. This observation confirms that the reported configuration is not an isolated solution, but rather representative of a broader set of effective slot distributions that satisfy the desired sidelobe performance and can be replicated by other researchers. The distribution in Fig. 11 also suggests that further optimization is possible. However, optimization is a complex task with numerous design parameters to consider, including the number of slot lengths, their distribution, acceptable SLL thresholds, the number of elements, and the ranges of incident and reflected angles. Therefore, we focused this work on demonstrating the potential of using slots for sensing and quantization lobe suppression, while reserving optimization for future research.

## Discussion

This paper presented a novel 1-bit HRIS that integrates AoA detection and beamforming capabilities while maintaining a simple and cost-effective design. The proposed HRIS leverages phase randomization to mitigate quantization lobes, improving beamforming efficiency despite using only 1-bit tunability and large element spacing. The sensing mechanism relies on randomly structured slots that couple a portion of the incident wave into a PPWG, where two coaxial connectors collect the signal. By applying computational processing techniques and an MLP, the system effectively estimates AoA with an accuracy of ±3$^{\circ }$, even under low-SNR conditions. Simulation results demonstrate that the proposed HRIS achieves robust beamforming performance while simultaneously detecting the propagation environment without requiring additional feedback loops. This low-complexity and power-efficient architecture makes the HRIS a promising candidate for applications in smart wireless communication, intelligent wireless power transfer configurations, and RF sensing systems.

There are several avenues to enhance the proposed HRIS. One area for improvement is the selection and optimization of slot sizes, which can enhance beamforming performance or increase power coupling at low SNR. Extending this configuration to detect and sense in 2D space while maintaining a simplified design is also an interesting direction to consider. Finally, fabricating this structure and experimentally verifying its functionality is a promising avenue currently under development.

## Data Availability

The data that support the findings of this study are available from the corresponding author upon reasonable request.

## Code availability

Code that supports the findings of this study is available from the corresponding author upon reasonable request.
